# Rare and Common Variants Associated with Alcohol Consumption Identify a Conserved Molecular Network

**DOI:** 10.1101/2024.02.26.582195

**Published:** 2024-03-01

**Authors:** Brittany S. Leger, John J. Meredith, Trey Ideker, Sandra Sanchez-Roige, Abraham A. Palmer

**Affiliations:** 1 Program in Biomedical Sciences, University of California San Diego, La Jolla, CA, USA; 2 Department of Psychiatry, University of California San Diego, La Jolla, CA, USA; 3 Department of Medicine, University of California San Diego, La Jolla, CA 92093, USA; 4 Institute for Genomic Medicine, University of California San Diego, La Jolla, CA 92093, USA; 5 Department of Medicine, Division of Genetic Medicine, Vanderbilt University, Nashville, TN, USA

## Abstract

Genome-wide association studies (GWAS) have identified hundreds of common variants associated with alcohol consumption. In contrast, rare variants have only begun to be studied for their role in alcohol consumption. No studies have examined whether common and rare variants implicate the same genes and molecular networks. To address this knowledge gap, we used publicly available alcohol consumption GWAS summary statistics (GSCAN, N=666,978) and whole exome sequencing data (Genebass, N=393,099) to identify a set of common and rare variants for alcohol consumption. Gene-based analysis of each dataset have implicated 294 (common variants) and 35 (rare variants) genes, including ethanol metabolizing genes *ADH1B* and *ADH1C,* which were identified by both analyses, and *ANKRD12*, *GIGYF1*, *KIF21B*, and *STK31*, which were identified only by rare variant analysis, but have been associated with related psychiatric traits. We then used a network colocalization procedure to propagate the common and rare gene sets onto a shared molecular network, revealing significant overlap. The shared network identified gene families that function in alcohol metabolism, including *ADH*, *ALDH*, *CYP*, and *UGT*. 74 of the genes in the network were previously implicated in comorbid psychiatric or substance use disorders, but had not previously been identified for alcohol-related behaviors, including *EXOC2*, *EPM2A*, *CACNB3*, and *CACNG4*. Differential gene expression analysis showed enrichment in the liver and several brain regions supporting the role of network genes in alcohol consumption. Thus, genes implicated by common and rare variants identify shared functions relevant to alcohol consumption, which also underlie psychiatric traits and substance use disorders that are comorbid with alcohol use.

## Introduction

Alcohol use disorder (**AUD**) is a highly heritable (Verhulst, Neale and Kendler, 2015) disease with a heavy public health burden (MacKillop et al., 2022). AUD can be viewed as the endpoint of a series of transitions, which begin with the initiation of use, regular alcohol consumption that continues with the escalation to hazardous drinking, and culminates in compulsive harmful use that persists despite negative consequences (Sanchez-Roige, Palmer and Clarke, 2020). As such, alcohol consumption is frequently studied as a proxy for AUD, as it is a component of AUD, and is a quantitative trait that is widely measured, providing large sample sizes for genetic studies. In particular, genome-wide association studies (**GWAS**) have identified numerous common variants that contribute to alcohol consumption, AUD, and related traits (Clarke et al., 2017; Liu et al., 2019; Saunders et al., 2022).

Recently, GWAS of other psychiatric disorders have extended their reach to rare variants. Variants with deleterious effects are under negative selection (Gibson, 2012), thus rare variants are predicted to have higher penetrance and higher effect sizes in disease than common variants (Kimura et al., 2021). Rare variants also tend to have developed more recently than common variants, leading to fewer variants being in linkage disequilibrium with rare variants. This makes the association of rare variants with genes less ambiguous, and increases the interpretability of rare variants, compared with common variants (Sazonovs and Barrett, 2018). Rare variant studies have revealed that these variants influence risk for multiple psychiatric disorders, including intellectual disability, autism spectrum disorder, and schizophrenia (Ganna et al., 2018; Charney et al., 2019; Antaki et al., 2022; Singh et al., 2022; Fu et al., 2023; Weiner et al., 2023). Because they are uncommon, rare variants are best identified using sequencing in conjunction with large sample sizes (Manolio et al., 2009; Backman et al., 2021; Wang et al., 2021; Karczewski et al., 2022). Although a few exome sequencing studies and rare variant studies for alcohol phenotypes have been undertaken (Vrieze et al., 2014; Marees et al., 2018; Curtis, 2022; Ahangari et al., 2023) the contribution of rare variation on alcohol behaviors remains poorly characterized, as does the relationship between common and rare variants.

One way to address the relationship between common and rare variants is by using biological knowledge networks. These networks contain information about the molecular interactions among genes and their products, both broadly and in disease contexts (Jia and Zhao, 2014; Farris, Harris and Ponomarev, 2015; Fong et al., 2019; Rosenthal et al., 2023). While the interplay between rare and common variant-implicated genes has been studied in network space for other psychiatric traits (Gilman et al., 2011; Ben-David and Shifman, 2012; Chang et al., 2018), it has not been studied for alcohol-related traits or other substance use disorders (**SUD**). Based on evidence from comparisons of common and rare variants for other psychiatric traits, we hypothesized that the same genes and molecular pathways would be identified by both approaches.

To test this hypothesis, we assembled data from UK Biobank (**UKB**) pertaining to both common and rare variants that are associated with alcohol consumption. We then used a network approach to investigate the biological overlap between common and rare variants for alcohol consumption. This approach allowed us to compare their relative contributions at the variant, gene, and molecular pathway levels.

## Results

### Common and Rare Variant Associations

**Figure 1.**
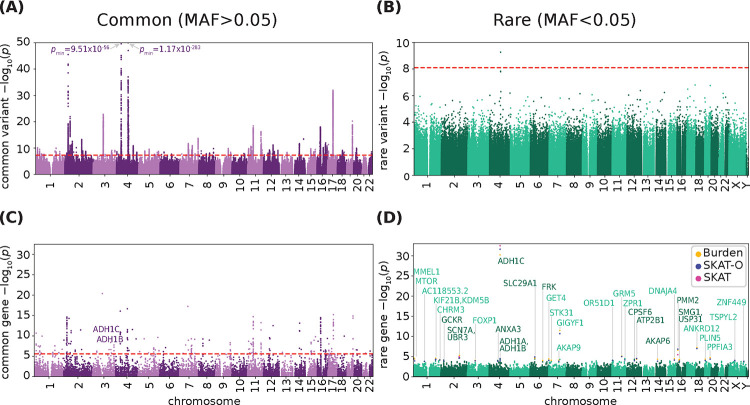
Common and rare variants mediate alcohol consumption. Manhattan plot of (**A**) common variants and (**B**) rare variants associated with alcohol consumption. Significance cutoff indicated in red (common: *p*<5×10−8; rare: *p*<8×10−9). p-value for peaks outside of range labeled. Rare variant MAC>2. (**C**) Manhattan plot of alcohol consumption common variant-implicated genes. Significance cutoff (*p*<2.6×10−6) indicated in red. Significant genes that overlap with rare-variant implicated genes are labeled. (**D**) Porcupine plot of genes calculated by burden test, SKAT-O, and SKAT algorithms from rare variants. Significantly associated genes (FDR<0.25) for each test are labeled and colored in yellow, blue, and pink, for burden, SKAT-O, and SKAT, respectively. See Figure S1a for individual manhattan plots for each test.

We obtained GWAS summary statistics from GSCAN, which recently performed a meta-analysis of alcohol consumption in Europeans (Saunders et al., 2022) (n=666,978, Figure 1A). 501 independent (r^2^) common (MAF>0.05) variants were significantly associated with alcohol consumption (*p*<5×10^−8^) (Saunders et al., 2022). Genome-wide significant rare variants were obtained from Genebass’s recent analysis of 393,099 individuals from the UKB (Karczewski et al., 2022) (Figure 1B). Three rare variants were significantly associated with alcohol consumption (*p*<8×10^−9^): one potential loss of function (pLoF) variant in *ADH1C*, a missense variant in *ADH1B*, and a synonymous mutation in *C4orf54* (Table S1). The mutations in *ADH1C* and *C4orf54* were both protective. *ADH1C* and *ADH1B* both have known roles in ethanol metabolism (Tolstrup et al., 2008; Le Daré, Lagente and Gicquel, 2019), but despite *C4orf54* being associated with substance use disorders in prior GWAS (Sollis et al., 2023), its function is poorly understood.

### Common and Rare Gene-Level Associations

Common loci were assigned to genes based on proximity using MAGMA (de Leeuw et al., 2015), identifying 294 genes (Figure 1C; Table S2, *p*<2.6×10^−6^). Rare variants were previously aggregated (Karczewski et al., 2022) into gene level associations using SKAT, SKAT-O, and a variant burden test (Karczewski et al., 2022). These tests identified four genes that were significantly correlated with alcohol consumption via both SKAT-O and burden tests (*p*_SKAT-O_<2.5×10^−7^; *p*_burden_<6.7×10^−7^): *ADH1C*, *PMM2*, *GIGYF1*, and *ANKRD12*. Only *ADH1C* was significantly associated by SKAT (*p*_SKAT_<2.5×10^−7^), and was the only gene previously associated with alcohol-related traits by common gene analysis.

We also considered a more lenient cutoff for genes from rare variants (FDR<0.25, Figure 1D, Figure S1A, Table S3), which identified 35 genes across all tests. 20 genes were identified by both SKAT-O and the burden test (Figure S1B), however, only *ADH1C* and *PMM2* were significant in all tests. 51% of genes were functionally annotated as loss of function, followed by missense and low confidence loss of function (40%), and the remaining 9% as synonymous (Figure S1C). 12 of these genes had previously been identified by common variants as mediating alcohol consumption and alcohol use traits in the GWAS catalog (Sollis et al., 2023) (Table S3; *p*=8.24×10^−33^, hypergeometric test). This includes alcohol dehydrogenase genes *ADH1A, ADH1B,* and *ADH1C*, and signaling genes *FOXP1, AKAP6, AKAP9, and GRM5*, highlighting the overlapping regulation of SUDs and psychiatric traits.

**Figure 2.**
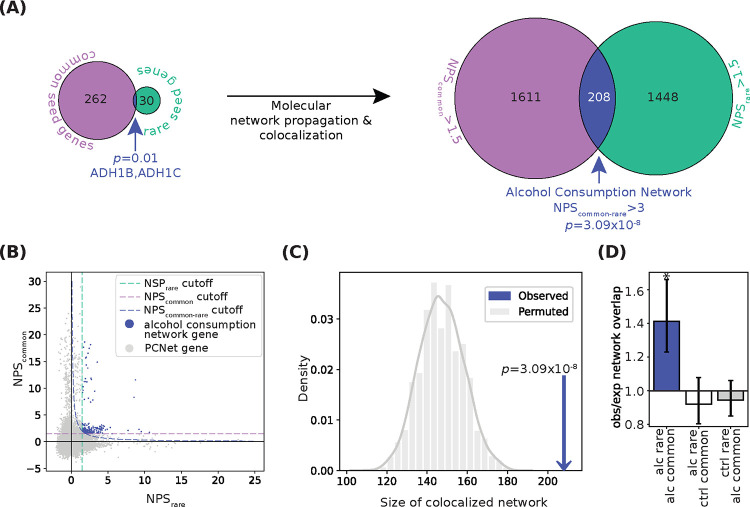
Convergence of rare and common variants on the network level. (**A**) Left, venn diagram showing overlap of common seed genes (purple) and rare seed genes (green). Overlapping genes are indicated in dark blue and labeled. Significance of overlap calculated via hypergeometric test. Right, Venn diagram of genes passing network proximity score (NPS) c of intersection indicated calculated in C. (**B**) NPS_common_ and NPS_rare_ for all genes in PCNet, with genes passing all thresholds for the alcohol consumption network (NPS_common-rare_ > 3, NPS_common_ > 1.5, and NPS_rare_ > 1.5) shown in dark blue. Dotted lines indicate NPS thresholds. (**C**) Observed (dark blue arrow) versus expected (gray distribution) size of the Alcohol Consumption Network following 10,000 permutations of NPS labels. p-value calculated via Z-test. (**D**) The observed-to-expected ratio of colocalized network size for networks calculated from common and rare seed genes from alcohol consumption and from control trait FEV1 (forced expiratory volume per second). Vertical bars indicate 95% confidence intervals. Significance calculated by Z-test, Bonferroni corrected. * indicates p=3.09×10−8. See also Figure S2C and Table S5 for additional controls.

*ADH1B* and *ADH1C* were identified by both the rare and common gene-based analyses (Figure 2A, *p*=0.01, hypergeometric test).

### Generation of the Alcohol Consumption Network

Next, we examined the molecular pathways wherein these alcohol consumption genes function (Figure 2A). We used the Parsimonious Composite Network (**PCNet**), a resource of 2.7 million pairwise associations among genes (Huang et al., 2018). PCNet is a consensus of 21 physical and functional interaction databases and integrates multiple lines of evidence, including protein-protein interactions, mRNA, protein co-expression across tissues, and literature curation.

We assigned network proximity scores (**NPS**) to each gene in PCNet using a random-walk algorithm, which computes the number of steps through the network to reach that gene from a set of seed genes. Seed genes from common and rare gene-set analyses were filtered for presence in PCNet, resulting in 264 common seed genes and 32 rare seed genes. NPS_common_ was calculated from common seed genes and NPS_rare_ was calculated from rare seed genes (Figure S2A). We then calculated the product of the two proximity scores to compute NPS_common-rare_ = NPS_common_ × NPS_rare_, and selected for high NPS_common_, NPS_rare_, and NPS_common-rare_ scores (Figure 2B). In this way, genes with the highest NPS_common-rare_ were close in the molecular network to both common and rare seed genes, even if they were not identified by the individual studies (Table S4).

We found that the alcohol consumption network contained significantly more genes (Figure 2C, *p*=3.09 × 10^−8^), and that the mean of NPS_common-rare_ was significantly higher than expected (Figure S2C, *p*=5.51 × 10^−6^). As a negative control, we produced networks using both the alcohol rare and common seed genes in conjunction with arbitrary traits; these negative controls did not produce networks that were larger than the permuted control (Figure 2D, Table S5). Additionally, when we considered a more stringent threshold for rare seed genes (*p*_SKAT-O_<2.5×10^−7^, n=4) we had similar results (Figure S2C). However, network colocalization was contingent upon *ADH1C*.

**Figure 3.**
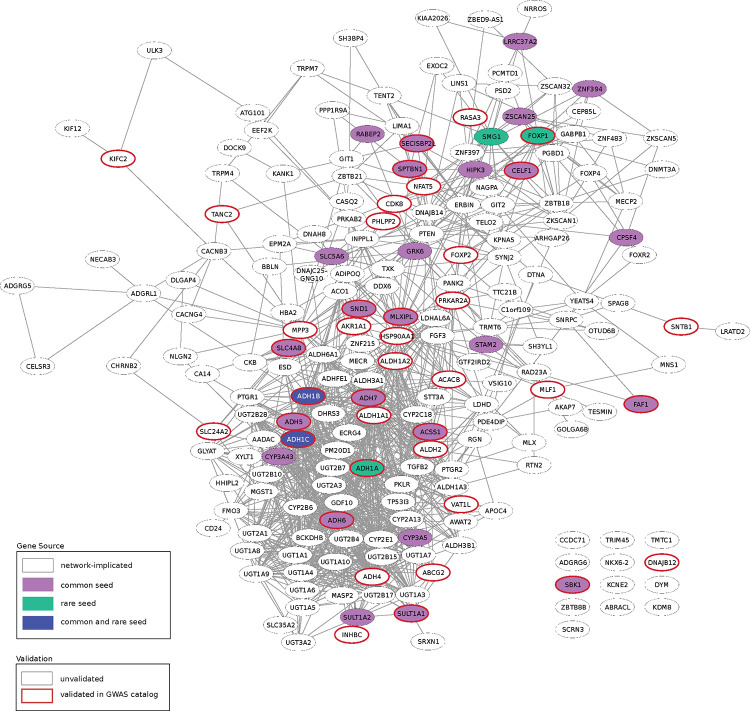
The Alcohol Consumption Network. Subnetwork of PCNet including all genes proximal to both rare and common alcohol consumption seed genes. Purple nodes indicate common seed genes, green nodes indicate rare seed genes, dark blue nodes indicate seeds in both sources, and white nodes are network-implicated genes. Edges maintained from PCNet. Red outlined nodes have previously been annotated in the GWAS catalog for alcohol use traits.

As shown in Figure 3, the alcohol consumption network contained 208 nodes, connected by 1,226 edges. 27 of 264 seed genes were maintained from the common seed genes. 5 of the 34 seed genes derived from rare variants were maintained into the network. *ADH1B* and *ADH1C*, which were seed genes for both common and rare, were both maintained into the network (Table S4).

### The Structure of the Alcohol Consumption Network

One of the goals of generating the network shown in Figure 3 is to identify the underlying biology identified by common and rare seed genes. Several gene families previously known to play a role in ethanol metabolism were present in the network (Figure S3). For example, 8 genes from the alcohol dehydrogenase (***ADH***) family (Le Daré, Lagente and Gicquel, 2019) and 7 aldehyde dehydrogenase (***ALDH***) family genes (Edenberg, 2007) are in the network. 6 cytochrome P450 (***CYP***) genes, which mediate about 10% of alcohol metabolism via the microsomal pathway (Hamitouche et al., 2006; Corella, 2012), were also in the network. In addition, genes from the non-oxidative ethanol metabolism pathways, which primarily function in phase II drug metabolism (Le Daré, Lagente and Gicquel, 2019), were also present. This includes 2 sulfotransferase (***SULT***) family genes, which metabolize ethanol into ethyl sulfate, and 18 genes in the UDP-Glycosyltransferase (***UGT***) superfamily, whose encoded proteins glucuronidate ethanol into ethyl glucuronide, a minor non-oxidative metabolite of ethanol (Walsham and Sherwood, 2014). Thus, the network recapitulates previously known biologies relevant to ethanol metabolism.

**Figure 4.**
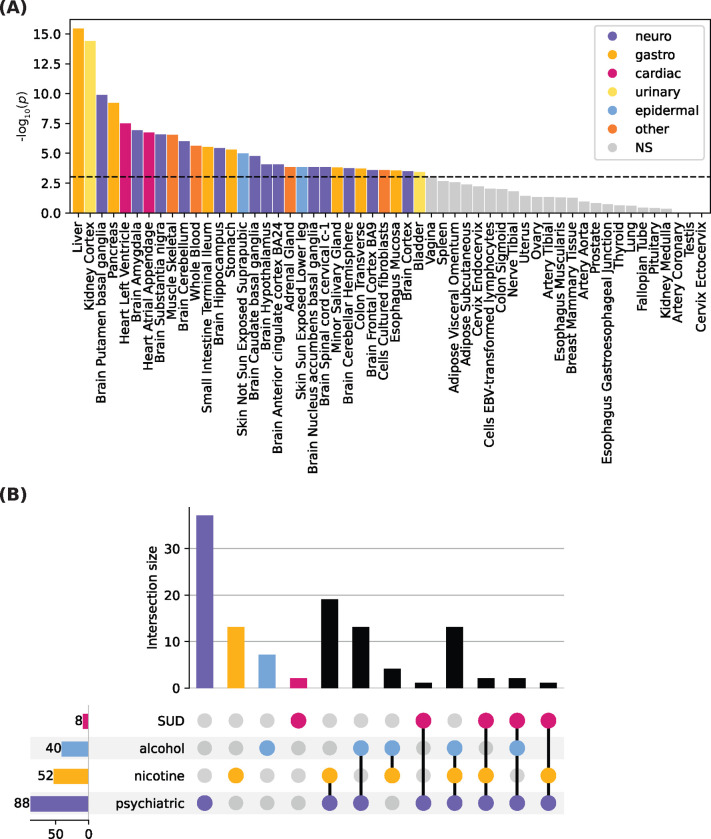
Validation of alcohol consumption network. (**A**) Enrichment of gene sets from the alcohol consumption network with bi-directional differential expression for 54 tissues from GTEX v8. Differentially expressed gene sets were defined by a two-sided t-tests per label, versus all remaining tissue types. Genes with *p*<9.26×10−4 (Bonferroni corrected) and absolute log fold change ≥ 0.58 are selected as differentially expressed. Significance was calculated as the probability of the hypergeometric test. Tissues are colored by type, non-significant (NS) associations are indicated in gray. (**B**) Upset plot showing the overlap of genes in the alcohol consumption network that have previously been annotated in the GWAS catalog for alcohol use traits, nicotine use and smoking traits, other substance use disorders (SUD), and psychiatric traits.

Another benefit of the network is the ability to identify relevant tissues. We found 25 tissues that were significantly enriched for differential gene expression (Figure 4A, Table S3), with high overlap of genes across tissues. Consistent with the presence of genes involved in ethanol metabolism in the network, the highest enrichment was in the liver and consisted of 115 genes, including 28 from the *ADH*, *ALDH*, *UGT*, *CYP*, and *SULT* families. In addition to the liver, numerous gastrointestinal tissues were also enriched: the gastrointestinal tract mediates absorption and gastric metabolism of alcohol, and chronic alcohol consumption may lead to inflammation and increased risk of gastrointestinal and esophageal cancers (Bode and Bode, 1997; Edenberg, 2007). As expected, all brain tissues were significantly enriched.

To determine whether the genes had been previously implicated by common variants in alcohol use, other SUDs, and related psychiatric disorders, we examined annotations from the GWAS catalog (Sollis et al., 2023). Specifically, we considered annotations for alcohol use, smoking and nicotine use, and other SUDs, including opioid, cannabis, and polysubstance use, and related psychiatric disorders (Figure 4B). 201 of the 208 genes in the network are annotated in the GWAS catalog. Of these, 40 have been previously associated in alcohol use (*p*=1.56 × 10^−3^, hypergeometric test) and 52 network genes in smoking traits (*p*=0.046, hypergeometric test) (Karlsson Linnér et al., 2021). 8 were identified for SUDs and 88 for psychiatric traits, though the enrichment was not significant for either. Of the genes associated with these traits, many had annotations in multiple categories, such as *EPM2A*, *EXOC2*, *NFAT5*, and *SNTB1*. These findings highlight the neuropsychiatric function of the network and point to a shared underlying mechanism across alcohol and polysubstance use.

Finally, to determine whether these genes had been previously implicated by rare variants in alcohol use, we examined gene-level annotations from GeneBass of genes in the network (Karczewski et al., 2022). 6 of the 208 network genes (*ADH1C*, *AKAP7*, *ATG101*, *DTNA*, *NKX6–2*, and *SYNJ2*) were associated with secondary alcohol use traits by rare variants, including use status and frequency of use, negative societal impacts from use, and alcoholic liver disease. Only *ADH1C* was also associated with alcohol use traits by common variants. Notably, these genes, excluding *ATG101*, were all associated with other SUDs and psychiatric traits through common variants.

## Discussion

The contribution of common variants in mediating alcohol consumption has been well documented, while rare variants represent a new frontier that has recently become feasible due to the availability of large scale sequencing data. Prior rare variant analysis identified 4 genes at a stringent (*p*_SKAT-O_<2.5×10^−7^) and 35 genes at a lenient threshold (FDR<0.25), demonstrating the importance of rare variants for alcohol-related behaviors (Figure 1). We combined the findings from common and rare variants to determine whether they identify convergent biological networks (Figure 2). We identified a highly significant network (Figure 3). The network emphasized the role of ethanol metabolism, which was further supported by the tissue specific enrichment in both brain and liver (Figure 4), consistent with decades of research on the genetics of alcohol consumption.

The role of common variants in ethanol metabolizing enzymes is well established for alcohol consumption and related traits (Sanchez-Roige, Palmer and Clarke, 2020; MacKillop et al., 2022). Similarly, rare variant analysis of alcohol consumption identified *ADH1A*, *ADH1B*, and *ADH1C*, which have well documented roles in ethanol metabolism (Edenberg, 2007). The network identified by the joint analysis of common and rare variants also identified genes for both oxidative and non-oxidative ethanol metabolism, including *ADH*, *ALDH*, *UGT*, *CYP*, and *SULT* family genes. Ethanol is primarily metabolized in the liver, but is also metabolized by the stomach and the brain (Zakhari, 2006), which was reflected in the high enrichment of network genes in the liver, gastrointestinal tissues, and the brain. Disulfiram, by inhibiting *ALDH1A1* - which was a gene in the alcohol consumption network - is an effective treatment for alcohol use disorder (Lanz et al., 2023), suggesting that other genes identified by our network could also be viable pharmacological targets.

In addition to ethanol metabolism, genes found by our analyses have also been associated with neuropsychiatric conditions that are correlated (Walters et al., 2018) and highly comorbid with alcohol use disorder, such as depression (Ribadier and Varescon, 2019), schizophrenia (Johnson et al., 2023), bipolar disorder (Grunze et al., 2021), neuroticism (Ribadier and Varescon, 2019), and cognitive dysfunction (Nunes et al., 2019). For example, the rare variant analysis identified *KIF21B* (Asselin et al., 2020), which has been associated with smoking initiation (Saunders et al., 2022), ADHD (Cross-Disorder Group of the Psychiatric Genomics Consortium, 2013), and schizophrenia (Trubetskoy et al., 2022). *GIGYF1* has been associated with Alzheimer’s disease (Burdett et al., no date) and schizophrenia (Ding et al., 2023). Finally, *SCN7A* has been associated with unipolar depression and educational attainment (Almomani et al., 2023). Similarly, the alcohol consumption network identified genes that have also been associated with neuropsychiatric conditions, such as genes from the FOXP family (i.e., *FOXP1, FOXP2*, *FOXP4* (Sollis et al., 2023)). Another example is *CACNB3* and *CACNG4*, calcium channel genes that have been associated with bipolar disorder and major depression((Sklar et al., 2012; Marshe et al., 2021). Finally, the gene *ADGRG6*, which was identified by the alcohol consumption network, has been associated with depression and smoking initiation (Sollis et al., 2023). Integrative analyses may help clarify the shared mechanisms of these conditions, but together this emphasizes shared genetic susceptibility across these traits.

While this study found that common and rare variants that were associated with alcohol consumption identified a shared network, there are several limitations to consider. We found that *ADH1C* is needed for network colocalization, showing that it is a hub gene for this network; this highlights the need for increased power of rare variants. We only studied alcohol consumption, however future study should also consider other AUD-relevant phenotypes. Similarly, methods for mapping common SNP to genes are imperfect; we used MAGMA but other more or less stringent methods might produce different results. Additionally, we used a lenient significance threshold to select rare variants (FDR>0.25), which likely introduced some false positives into the network analysis. However, we repeated this analysis with a more stringent cutoff for rare variants (*p*_SKAT-O_<2.5×10^−7^) and found little change in significance of network overlap. Additionally, NetColoc is robust to false positives, but functions best with a moderate number of input genes (Rosenthal et al., 2023).

While future improvements to our methodology and the underlying data will improve our ability to understand rare and common variant interaction, this work identified the first gene network from common and rare variants of alcohol consumption.

## Materials and Methods

### Lead Contact

Further information and requests for resources should be directed to aapalmer@ucsd.edu and sanchezroige@health.ucsd.edu.

### Materials availability

This study did not generate new unique reagents.

### Data and code availability

All code used for analysis and data visualization is freely available in public repositories. All original code is publicly available at https://github.com/BSLeger/rare_common_alcohol_comparison.

Any additional information required to reanalyze the data reported in this paper is available from the lead contact upon request.

### Data acquisition

#### Common variant experimental and control data acquisition

The common variant summary statistics for alcohol consumption were obtained from the GWAS & Sequencing Consortium of Alcohol and Nicotine use. Summary statistics were computed via a meta-analysis of GWAS results representing 666,978 individuals of European ancestry (Saunders et al., 2022). Summary statistics for common variant negative control traits were obtained from the Neale Lab Round 2 GWAS (Abbott et al., 2022) (http://www.nealelab.is/uk-biobank). Phenotype codes are FEV1: Forced Expiratory Volume per Second (20153_irnt) and Heel Quantitative Ultrasound Index (QUI) (4104_irnt). These negative control traits were selected as they have similar numbers of implicated genes to alcohol consumption (250<N<350), similar SNP heritability to alcohol consumption (*h*^*2*^ >0.30), and minimal genetic correlation with a comparable alcohol consumption trait (Amount of Alcohol Drunk on a Typical Drinking Day (20403)) (|*r*_g_| < 0.38). These estimates were obtained from the UKB Heritability browser (https://nealelab.github.io/UKBB_ldsc/h2_browser.html) and UKB Genetic Correlation browser (https://ukbb-rg.hail.is/), both generated by the Neale Lab (Abbott et al., 2022).

#### Rare variant and gene experimental and control data acquisition and filtering

Rare variant data was downloaded from Genebass’s Hail library (gs://ukbb-exome-public/500k/results/variant_results.mt), and queried for alcohol consumption by phenotype code (alcohol_intake_custom) using Hail. To increase the confidence in rare variants, we selected for alcohol consumption rare variants that have MAC in the top 50% (MAC>2). Rare variant gene-level associations was downloaded from Genebass browser (https://app.genebass.org/). Due to limited comparisons between rare variant datasets, rare variant controls were filtered based on heritability and genetic correlations listed above, which were calculated based on common variants. Rare variant controls were chosen if they had heritability greater than 0 (*h*^*2*^ >0.01), minimal genetic correlation with a comparable alcohol consumption trait (Amount of Alcohol Drunk on a Typical Drinking Day (20403)) (0.0< |*r*_g_| < 0.2), and had the minimum number of rare seed genes recommended for network propagation using NetColoc (n>5), using comparable significance cutoffs as used for alcohol consumption rare seed genes (false discovery rate corrected burden or SKAT-O or SKAT<0.25, calculated for each individual dataset). Phenotype codes are as follows: Alcohol Consumption (alcohol_intake_custom), FEV1: forced expiratory volume per second (20153), Pulse Rate (4194), Heel bone mineral density (BMD) T-score, automated (78), Other malignant neoplasms of skin (C44), Malignant neoplasm of breast (C50). The stringent alcohol consumption rare seed genes were selected if the genes were significant by any test (*p*<0.05) after bonferroni correction (*p*_SKAT-O_<2.5×10^−7^; *p*_burden_<6.7×10^−7^, *p*_SKAT_<2.5×10^−7^). Genes were considered leniently significant if any of the gene tests identified the genes as significant (*p*<0.25) after false discovery rate correction (*p*_SKAT-O_<1.5×10^−4^; *p*_burden_<1.1×10^−4^, *p*_SKAT_<2.7×10^−5^).

#### Molecular Interaction Networks

The Parsimonious Composite Network (Huang et al., 2018) (PCNet v1.4) was obtained from the network data exchange (NDEx, ndexbio.org), UUID: c3554b4e-8c81-11ed-a157-005056ae23aa. PCNet is a molecular interaction resource formed from integrating 21 interaction databases that contain various evidence types, including physical protein-protein, genetic, co-expression, and co-citation evidence. Each interaction in PCNet is supported by at least two of the component databases, a threshold chosen to maximize the ability of PCNet to perform gene set recovery tasks via network propagation. All seed genes were mapped to the nodes of PCNet via gene symbols.

### Common variant gene mapping

We generated gene-level significance values from the SNP-level summary statistics using the MAGMA algorithm (de Leeuw et al., 2015) using default parameters. Annotation windows were 10 kb, and the 1000 Genomes European reference panel was used for genome , and Hg38 Gene locations, downloaded from MAGMA’s launch page (https://ctg.cncr.nl/software/magma). MAGMA projects the SNP matrix onto principal components, and uses the principal components to predict for the phenotype using linear regression. Association of the gene to the phenotype using the principal component SNP matrix is used to calculate an F statistic, which is used to calculate the *p*-value for the individual genes. Genes were considered significant if they were *p*<2.6×10^−6^.

### Generation of the alcohol consumption network

#### Network propagation and co-localization

We used the Python package NetColoc (Rosenthal et al., 2023) (https://pypi.org/project/netcoloc/) for network propagation and co-localization. The sets of significant trait-associated genes from GWAS were used as “seed” genes for network propagation using a Random Walk with Restart (Vanunu et al., 2010) algorithm. Following network propagation with α=0.5, we calculated a network proximity score (NPS) for each gene in the network by comparing the observed results to a null distribution. The null distribution was formed by propagating 1,000 randomly selected seed gene sets. Each set was sampled to preserve the size and degree distribution of the original input set. As previously implemented (Rosenthal et al., 2021, 2023; Wright et al., 2023), we binned all genes in the network by degree with a minimum of 10 nodes per bin. For each gene, the NPS was calculated as a z-score comparing the observed heat at that gene after network propagation of the gene set, to the mean of the null distribution heats at that gene. All heat values are log-transformed to ensure the distributions are approximately normal.

NetColoc recommends fewer than 500 input seed genes given the sample space of PCNet (~18,000 genes). Therefore, we employed a weighted sampling procedure for any trait having more than 500 significantly associated genes. We sampled 500 genes from the set of all significant genes (weighted by −log_10_p from GWAS) and ran the propagation analysis from this subset. After 100 repetitions, the 75% percentile NPS score was selected to approximate a consensus score for each gene.

From input seed genes from common and rare variants, we independently calculated $NPS_{\text{common}}$ and $NPS_{\text{rare}}$ for each trait. We then defined a gene as colocalized between both if it had high proximity to both input sets. Therefore, we defined the combined network proximity $NPS_{\text{common-rare}}$ as the product of the independent dataset vectors:

$$NPS_{\mathrm{common-rare}}=NPS_{\mathrm{common}}*NPS_{\mathrm{rare}}$$

#### Definition of the alcohol consumption network

From NPS_common-rare,_ we selected genes with high proximity scores from both common and rare sources to define the network using the following thresholds: NPS_common-rare_ > 3, NPS_common_ > 1.5, and NPS_rare_ > 1.5. To calculate the significance of the network co-localization, we compared the conserved network size and the mean NPS_common-rare_ to a permuted null distribution. We permuted the labels of NPS_rare_ and NPS_common_ 10,000 times, and each time calculated the mean NPS_common-rare_ across all genes and the number of genes passing the above thresholds. For genes present in both input sets, labels were permuted separately to maintain the higher expected distribution for these genes. The significance of the conserved network size and mean NPS_common-rare_ was calculated by Z-test.

### Validation and functional annotation

#### Gene family annotation

Gene families were manually assigned based on gene families identified from the Uniprot ID mapping function (https://www.uniprot.org/id-mapping) on 28 November 2023. Families were assigned broadly to make functional groups more evident, with a minimum of 2 genes per family required to be labeled.

#### GWAS catalog

To identify previously annotated genes, we used GWAS findings aggregated by the GWAS catalog (https://www.ebi.ac.uk/gwas/). The GWAS catalog’s gene level associations v.1.0.2 were downloaded on 2 August 2023. We identified genes that had previously been associated with various traits by querying the Mapped Trait and the Disease/Trait for various keywords (see github for specific parameters). Traits were grouped into alcohol use traits, smoking and nicotine use traits, non-alcohol or smoking substance use disorders (for example, opioid use disorder), and non-SUD neurological and psychiatric traits (including cognitive traits, mental health and psychiatric traits, and neuro-degenerative traits). All groups are mutually exclusive. Within each group, traits are listed as Mapped Trait: Disease/Trait for clarity, and listed only once per gene for readability. Enrichment for each group was calculated using a hypergeometric test. Genes mentioned in text were reconfirmed using the GWAS catalog browser.

#### Rare Variant PheWAS

To assess the association of network genes with other phenotypes through rare variants, gene level PheWAS results were downloaded from Genebass’s Hail database (gs://ukbb-exome-public/500k/results/results.mt). Phenotypes were mapped to network genes by gene symbol. Phenotypes were determined as significant using the same *p*-value cutoffs as used for lenient seed genes from alcohol consumption (*p*_SKAT-O_<1.5×10^−4^; *p*_burden_<1.1×10^−4^, *p*_SKAT_<2.7×10^−5^).

#### Tissue Enrichment

To assess the tissue-specific expression of network genes, we used the Functional Mapping and Annotation of Genome-Wide Association Studies (FUMA) suite’s gene to function tool (Watanabe et al., 2017). We used FUMA to calculate the enrichment of gene sets for 54 tissue types from human GTEx v8 (GTEx Consortium, 2020). As described previously, this method takes normalized gene expressions (reads per kilobase per million, RPKM) from each GTEx tissue, and maps these genes to entrez ID (Watanabe et al., 2017). Precalculated differentially expressed genes (DEG) sets were defined using a two-sided t-test per label versus all remaining tissue types. Genes with a Bonferroni corrected *p*-value<0.05 and absolute log fold change≥0.58 were selected as DEG. For the signed DEG, the direction of expression was taken into account. The −log 10(*p*-values) in the graph were calculated by hypergeometric test (Watanabe et al., 2017).

## Supplementary Material

Supplement 1

Supplement 2

## References

[R1] Abbott L. (2022) Nealelab/UKBB_ldsc: v2.0.0 (Round 2 GWAS update). Available at: 10.5281/zenodo.7186871.

[R2] Ahangari M. (2023) ‘Improving the discovery of rare variants associated with alcohol problems by leveraging machine learning phenotype prediction and functional information’, bioRxiv. Available at: 10.1101/2023.09.11.557163.

[R3] Almomani R. (2023) ‘Genetic Profiling of Sodium Channels in Diabetic Painful and Painless and Idiopathic Painful and Painless Neuropathies’, International journal of molecular sciences, 24(9). Available at: 10.3390/ijms24098278. PMC10179245 37175987

[R4] Antaki D. (2022) ‘A phenotypic spectrum of autism is attributable to the combined effects of rare variants, polygenic risk and sex’, Nature genetics, 54(9), pp. 1284–1292. 35654974 10.1038/s41588-022-01064-5 PMC9474668

[R5] Asselin L. (2020) ‘Mutations in the KIF21B kinesin gene cause neurodevelopmental disorders through imbalanced canonical motor activity’, Nature communications, 11(1), p. 2441. 10.1038/s41467-020-16294-6 PMC7229210 32415109

[R6] Backman J.D. (2021) ‘Exome sequencing and analysis of 454,787 UK Biobank participants’, Nature, 599(7886), pp. 628–634. 34662886 10.1038/s41586-021-04103-z PMC8596853

[R7] Ben-David E. and Shifman S. (2012) ‘Networks of neuronal genes affected by common and rare variants in autism spectrum disorders’, PLoS genetics, 8(3), p. e1002556. 22412387 10.1371/journal.pgen.1002556 PMC3297570

[R8] Bode C. and Bode J.C. (1997) ‘Alcohol’s role in gastrointestinal tract disorders’, Alcohol health and research world, 21(1), pp. 76–83. 15706765 PMC6826790

[R9] Burdett T. (no date) GWAS Catalog. Available at: https://www.ebi.ac.uk/gwas/publications/34446935 (Accessed: 19 September 2023).

[R10] Chang X. (2018) ‘Common and Rare Genetic Risk Factors Converge in Protein Interaction Networks Underlying Schizophrenia’, Frontiers in genetics, 9, p. 434. 30323833 10.3389/fgene.2018.00434 PMC6172705

[R11] Charney A.W. (2019) ‘Contribution of Rare Copy Number Variants to Bipolar Disorder Risk Is Limited to Schizoaffective Cases’, Biological psychiatry, 86(2), pp. 110–119. 30686506 10.1016/j.biopsych.2018.12.009 PMC6586545

[R12] Clarke T.-K. (2017) ‘Genome-wide association study of alcohol consumption and genetic overlap with other health-related traits in UK Biobank (N=112 117)’, Molecular psychiatry, 22(10), pp. 1376–1384. 28937693 10.1038/mp.2017.153 PMC5622124

[R13] Corella D. (2012) ‘Alcohol intake’, Progress in molecular biology and translational science, 108, pp. 261–292. 22656381 10.1016/B978-0-12-398397-8.00011-3

[R14] Cross-Disorder Group of the Psychiatric Genomics Consortium (2013) ‘Identification of risk loci with shared effects on five major psychiatric disorders: a genome-wide analysis’, The Lancet, 381(9875), pp. 1371–1379. 10.1016/S0140-6736(12)62129-1 PMC3714010 23453885

[R15] Curtis D. (2022) ‘Investigation of Association of Rare, Functional Genetic Variants With Heavy Drinking and Problem Drinking in Exome Sequenced UK Biobank Participants’, Alcohol and alcoholism , 57(4), pp. 421–428. 33893496 10.1093/alcalc/agab031 PMC9270990

[R16] Ding Z. (2023) ‘Genetic Ablation of GIGYF1, Associated With Autism, Causes Behavioral and Neurodevelopmental Defects in Zebrafish and Mice’, Biological psychiatry, 94(10), pp. 769–779. 36924980 10.1016/j.biopsych.2023.02.993 PMC10502190

[R17] Edenberg H.J. (2007) ‘The genetics of alcohol metabolism: role of alcohol dehydrogenase and aldehyde dehydrogenase variants’, Alcohol research & health: the journal of the National Institute on Alcohol Abuse and Alcoholism, 30(1), pp. 5–13. 17718394 PMC3860432

[R18] Farris S.P., Harris R.A. and Ponomarev I. (2015) ‘Epigenetic modulation of brain gene networks for cocaine and alcohol abuse’, Frontiers in neuroscience, 9, p. 176. 26041984 10.3389/fnins.2015.00176 PMC4438259

[R19] Fong S.H. (2019) ‘Strategies for Network GWAS Evaluated Using Classroom Crowd Science’, Cell systems, 9(4), p. 414. 31647918 10.1016/j.cels.2019.09.001

[R20] Fu S. (2023) ‘Autism-specific PTEN p.Ile135Leu variant and an autism genetic background combine to dysregulate cortical neurogenesis’, American journal of human genetics, 110(5), pp. 826–845. 37098352 10.1016/j.ajhg.2023.03.015 PMC10183467

[R21] Ganna A. (2018) ‘Quantifying the Impact of Rare and Ultra-rare Coding Variation across the Phenotypic Spectrum’, American journal of human genetics, 102(6), pp. 1204–1211. 29861106 10.1016/j.ajhg.2018.05.002 PMC5992130

[R22] Gibson G. (2012) ‘Rare and common variants: twenty arguments’, Nature reviews. Genetics, 13(2), pp. 135–145. 10.1038/nrg3118 PMC4408201 22251874

[R23] Gilman S.R. (2011) ‘Rare de novo variants associated with autism implicate a large functional network of genes involved in formation and function of synapses’, Neuron, 70(5), pp. 898–907. 21658583 10.1016/j.neuron.2011.05.021 PMC3607702

[R24] Grunze H. (2021) ‘Comorbid Bipolar and Alcohol Use Disorder-A Therapeutic Challenge’, Frontiers in psychiatry / Frontiers Research Foundation, 12, p. 660432. 10.3389/fpsyt.2021.660432 PMC8021702 33833701

[R25] Consortium GTEx (2020) ‘The GTEx Consortium atlas of genetic regulatory effects across human tissues’, Science, 369(6509), pp. 1318–1330. 32913098 10.1126/science.aaz1776 PMC7737656

[R26] Hamitouche S. (2006) ‘Ethanol oxidation into acetaldehyde by 16 recombinant human cytochrome P450 isoforms: role of CYP2C isoforms in human liver microsomes’, Toxicology letters, 167(3), pp. 221–230. 17084997 10.1016/j.toxlet.2006.09.011

[R27] Huang J.K. (2018) ‘Systematic Evaluation of Molecular Networks for Discovery of Disease Genes’, Cell systems, 6(4), pp. 484–495.e5. 29605183 10.1016/j.cels.2018.03.001 PMC5920724

[R28] Jia P. and Zhao Z. (2014) ‘Network.assisted analysis to prioritize GWAS results: principles, methods and perspectives’, Human genetics, 133(2), pp. 125–138. 24122152 10.1007/s00439-013-1377-1 PMC3943795

[R29] Johnson E.C. (2023) ‘Investigation of convergent and divergent genetic influences underlying schizophrenia and alcohol use disorder’, Psychological medicine, 53(4), pp. 1196–1204. 34231451 10.1017/S003329172100266X PMC8738774

[R30] Karczewski K.J. (2022) ‘Systematic single-variant and gene-based association testing of thousands of phenotypes in 394,841 UK Biobank exomes’, Cell genomics, 2(9), p. 100168. 36778668 10.1016/j.xgen.2022.100168 PMC9903662

[R31] Karlsson Linnér R. (2021) ‘Multivariate analysis of 1.5 million people identifies genetic associations with traits related to self-regulation and addiction’, Nature neuroscience, pp. 1–10. 10.1038/s41593-021-00908-3 PMC8484054 34446935

[R32] Kimura H. (2021) ‘Elucidation of molecular pathogenesis and drug development for psychiatric disorders from rare disease-susceptibility variants’, Neuroscience research, 170, pp. 24–31. 33316300 10.1016/j.neures.2020.11.008

[R33] Lanz J. (2023) ‘Disulfiram: Mechanisms, Applications, and Challenges’, Antibiotics (Basel, Switzerland), 12(3). Available at: 10.3390/antibiotics12030524. PMC10044060 36978391

[R34] Le Daré B., Lagente V. and Gicquel T. (2019) ‘Ethanol and its metabolites: update on toxicity, benefits, and focus on immunomodulatory effects’, Drug metabolism reviews, 51(4), pp. 545–561. 31646907 10.1080/03602532.2019.1679169

[R35] de Leeuw C.A. (2015) ‘MAGMA: generalized gene-set analysis of GWAS data’, PLoS computational biology, 11(4), p. e1004219. 25885710 10.1371/journal.pcbi.1004219 PMC4401657

[R36] Liu M. (2019) ‘Association studies of up to 1.2 million individuals yield new insights into the genetic etiology of tobacco and alcohol use’, Nature genetics, 51(2), pp. 237–244. 30643251 10.1038/s41588-018-0307-5 PMC6358542

[R37] MacKillop J. (2022) ‘Hazardous drinking and alcohol use disorders’, Nature reviews. Disease primers, 8(1), p. 80. 10.1038/s41572-022-00406-1 PMC10284465 36550121

[R38] Manolio T.A. (2009) ‘Finding the missing heritability of complex diseases’, Nature, 461(7265), pp. 747–753. 19812666 10.1038/nature08494 PMC2831613

[R39] Marees A.T. (2018) ‘Exploring the role of low-frequency and rare exonic variants in alcohol and tobacco use’, Drug and alcohol dependence, 188, pp. 94–101. 29758381 10.1016/j.drugalcdep.2018.03.026

[R40] Marshe V.S. (2021) ‘Genome-wide analysis suggests the importance of vascular processes and neuroinflammation in late-life antidepressant response’, Translational psychiatry, 11(1), p. 127. 33589590 10.1038/s41398-021-01248-3 PMC7884410

[R41] Nunes P.T. (2019) ‘Aging with alcohol-related brain damage: Critical brain circuits associated with cognitive dysfunction’, International review of neurobiology, 148, pp. 101–168. 31733663 10.1016/bs.irn.2019.09.002 PMC7372724

[R42] Ribadier A. and Varescon I. (2019) ‘Anxiety and depression in alcohol use disorder individuals: the role of personality and coping strategies’, Substance use & misuse, 54(9), pp. 1475–1484. 30973041 10.1080/10826084.2019.1586950

[R43] Rosenthal S.B. (2021) ‘A convergent molecular network underlying autism and congenital heart disease’, Cell systems, 12(11), pp. 1094–1107.e6. 34411509 10.1016/j.cels.2021.07.009 PMC8602730

[R44] Rosenthal S.B. (2023) ‘Mapping the common gene networks that underlie related diseases’, Nature protocols, 18(6), pp. 1745–1759. 36653526 10.1038/s41596-022-00797-1 PMC10257754

[R45] Sanchez-Roige S., Palmer A.A. and Clarke T.-K. (2020) ‘Recent Efforts to Dissect the Genetic Basis of Alcohol Use and Abuse’, Biological psychiatry, 87(7), pp. 609–618. 31733789 10.1016/j.biopsych.2019.09.011 PMC7071963

[R46] Saunders G.R.B. (2022) ‘Genetic diversity fuels gene discovery for tobacco and alcohol use’, Nature, 612(7941), pp. 720–724. 36477530 10.1038/s41586-022-05477-4 PMC9771818

[R47] Sazonovs A. and Barrett J.C. (2018) ‘Rare-Variant Studies to Complement Genome-Wide Association Studies’, Annual review of genomics and human genetics, 19, pp. 97–112. 10.1146/annurev-genom-083117-021641 29801418

[R48] Singh T. (2022) ‘Rare coding variants in ten genes confer substantial risk for schizophrenia’, Nature, 604(7906), pp. 509–516. 35396579 10.1038/s41586-022-04556-w PMC9805802

[R49] Sklar P. (2012) ‘Large-scale genome-wide association analysis of bipolar disorder identifies a new susceptibility locus near ODZ4’, Nature genetics, 44(9), p. 1072. 10.1038/ng.943 PMC3637176 21926972

[R50] Sollis E. (2023) ‘The NHGRI-EBI GWAS Catalog: knowledgebase and deposition resource’, Nucleic acids research, 51(D1), pp. D977–D985. 36350656 10.1093/nar/gkac1010 PMC9825413

[R51] Tolstrup J.S. (2008) ‘Alcoholism and alcohol drinking habits predicted from alcohol dehydrogenase genes’, The pharmacogenomics journal, 8(3), pp. 220–227. 17923853 10.1038/sj.tpj.6500471

[R52] Trubetskoy V. (2022) ‘Mapping genomic loci implicates genes and synaptic biology in schizophrenia’, Nature, 604(7906), pp. 502–508. 35396580 10.1038/s41586-022-04434-5 PMC9392466

[R53] Vanunu O. (2010) ‘Associating genes and protein complexes with disease via network propagation’, PLoS computational biology, 6(1), p. e1000641. 20090828 10.1371/journal.pcbi.1000641 PMC2797085

[R54] Verhulst B., Neale M.C. and Kendler K.S. (2015) ‘The heritability of alcohol use disorders: a meta-analysis of twin and adoption studies’, Psychological medicine, 45(5), pp. 1061–1072. 25171596 10.1017/S0033291714002165 PMC4345133

[R55] Vrieze S.I. (2014) ‘Rare nonsynonymous exonic variants in addiction and behavioral disinhibition’, Biological psychiatry, 75(10), pp. 783–789. 24094508 10.1016/j.biopsych.2013.08.027 PMC3975816

[R56] Walsham N.E. and Sherwood R.A. (2014) ‘Chapter Two - Ethyl Glucuronide and Ethyl Sulfate’, in Makowski (ed.) Advances in Clinical Chemistry. Elsevier, pp. 47–71. 10.1016/bs.acc.2014.09.006 25735859

[R57] Walters R.K. (2018) ‘Transancestral GWAS of alcohol dependence reveals common genetic underpinnings with psychiatric disorders’, Nature neuroscience, 21(12), pp. 1656–1669. 30482948 10.1038/s41593-018-0275-1 PMC6430207

[R58] Wang Q. (2021) ‘Rare variant contribution to human disease in 281,104 UK Biobank exomes’, Nature, 597(7877), pp. 527–532. 34375979 10.1038/s41586-021-03855-y PMC8458098

[R59] Watanabe K. (2017) ‘Functional mapping and annotation of genetic associations with FUMA’, Nature communications, 8(1), p. 1826. 10.1038/s41467-017-01261-5 PMC5705698 29184056

[R60] Weiner D.J. (2023) ‘Polygenic architecture of rare coding variation across 394,783 exomes’, Nature, 614(7948), pp. 492–499. 36755099 10.1038/s41586-022-05684-z PMC10614218

[R61] Wright S.N. (2023) ‘Genome-wide association studies of human and rat BMI converge on synapse, epigenome, and hormone signaling networks’, Cell reports, 42(8), p. 112873. 37527041 10.1016/j.celrep.2023.112873 PMC10546330

[R62] Zakhari S. (2006) ‘Overview: how is alcohol metabolized by the body?’, Alcohol research & health: the journal of the National Institute on Alcohol Abuse and Alcoholism, 29(4), pp. 245–254. 17718403 PMC6527027

