# Supplementary material for: Rare and Common Variants Associated with Alcohol Consumption Identify a Conserved Molecular Network
Source: bioRxiv. 2024 Mar 1:2024.02.26.582195. Preprint. [Version 1] doi: 10.1101/2024.02.26.582195 (PMC10925118; doi:10.1101/2024.02.26.582195)
Supplement: Supplement 2 [file NIHPP2024.02.26.582195v1-supplement-2.pdf]

bioRxiv preprint doi: https://doi.org/10.1101/2024.02.26.582195; this version posted March 1, 2024. The copyright holder for this preprint (which was not certified by peer review) is the author/funder, who has granted bioRxiv a license to display the preprint in perpetuity. It is made available under aCC-BY 4.0 International license.

Supplemental Figures

bioRxiv preprint doi: https://doi.org/10.1101/2024.02.26.582195; this version posted March 1, 2024. The copyright holder for this preprint (which was not certified by peer review) is the author/funder, who has granted bioRxiv a license to display the preprint in perpetuity. It is made available under aCC-BY 4.0 International license.

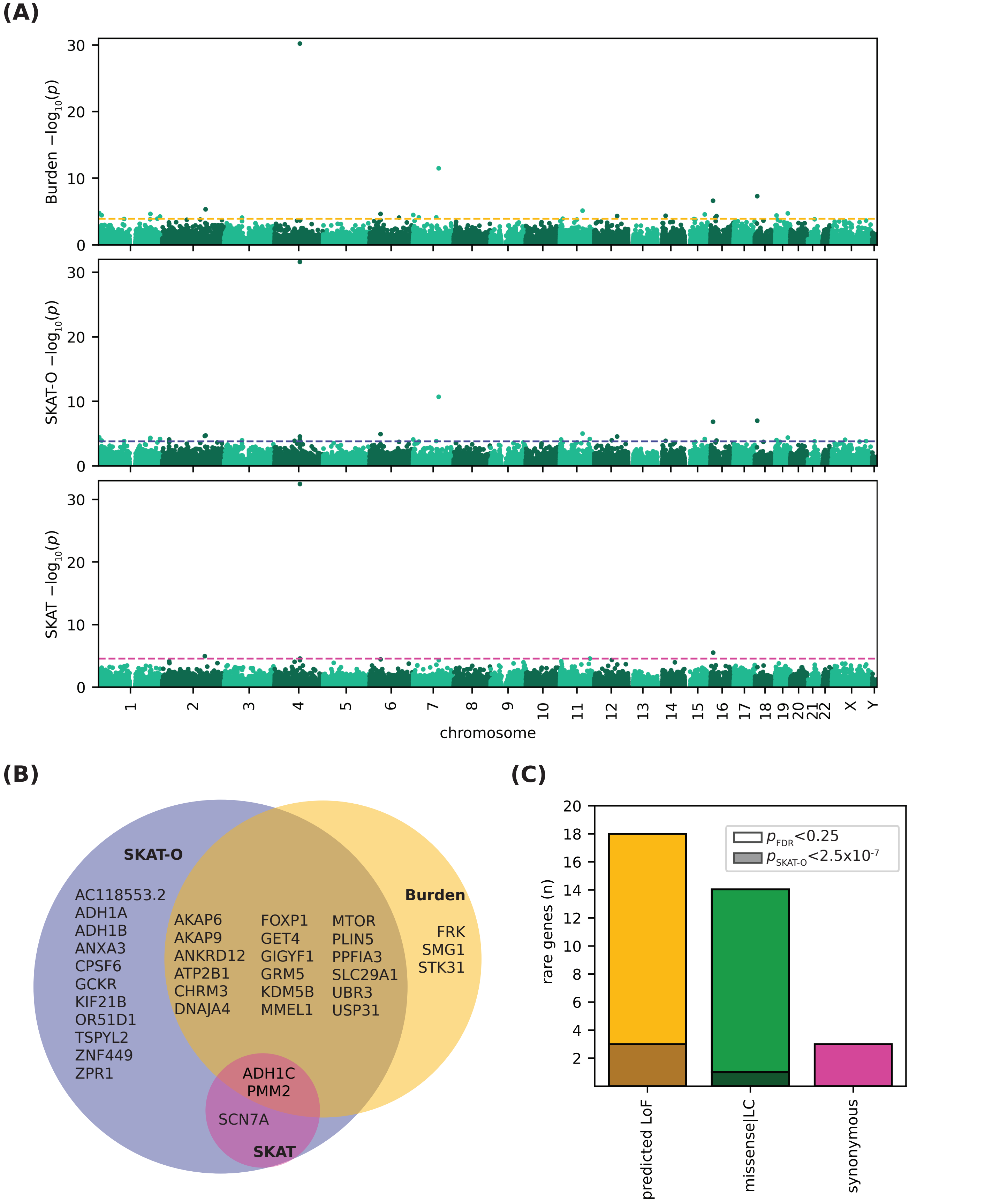

Supplemental Figure 1. Rare variants-implicated genes mediating alcohol consumption. (**A**) Manhattan plot of association with alcohol consumption for rare-variant implicated genes calculated by burden test (top), SKAT-O (middle), and SKAT (bottom). Dotted lines indicate FDR<0.25 cutoff for each test. (**B**) Venn diagram of leniently significant (FDR<0.25) genes identified from rare variants for alcohol consumption, broken down by SNP to gene algorithm used (burden, SKAT, SKAT-O). (**C**) Stacked bar chart of rare seed gene mutation type, grouped by SNP to gene algorithm. FDR<0.25 genes are shown in light colors, and $p_{SKAT\text{-}O}$<2.5x10-7 genes are shown in dark colors. No $p_{SKAT\text{-}O}$<2.5x10-7 genes were annotated as synonymous.

bioRxiv preprint doi: https://doi.org/10.1101/2024.02.26.582195; this version posted March 1, 2024. The copyright holder for this preprint (which was not certified by peer review) is the author/funder, who has granted bioRxiv a license to display the preprint in perpetuity. It is made available under aCC-BY 4.0 International license.

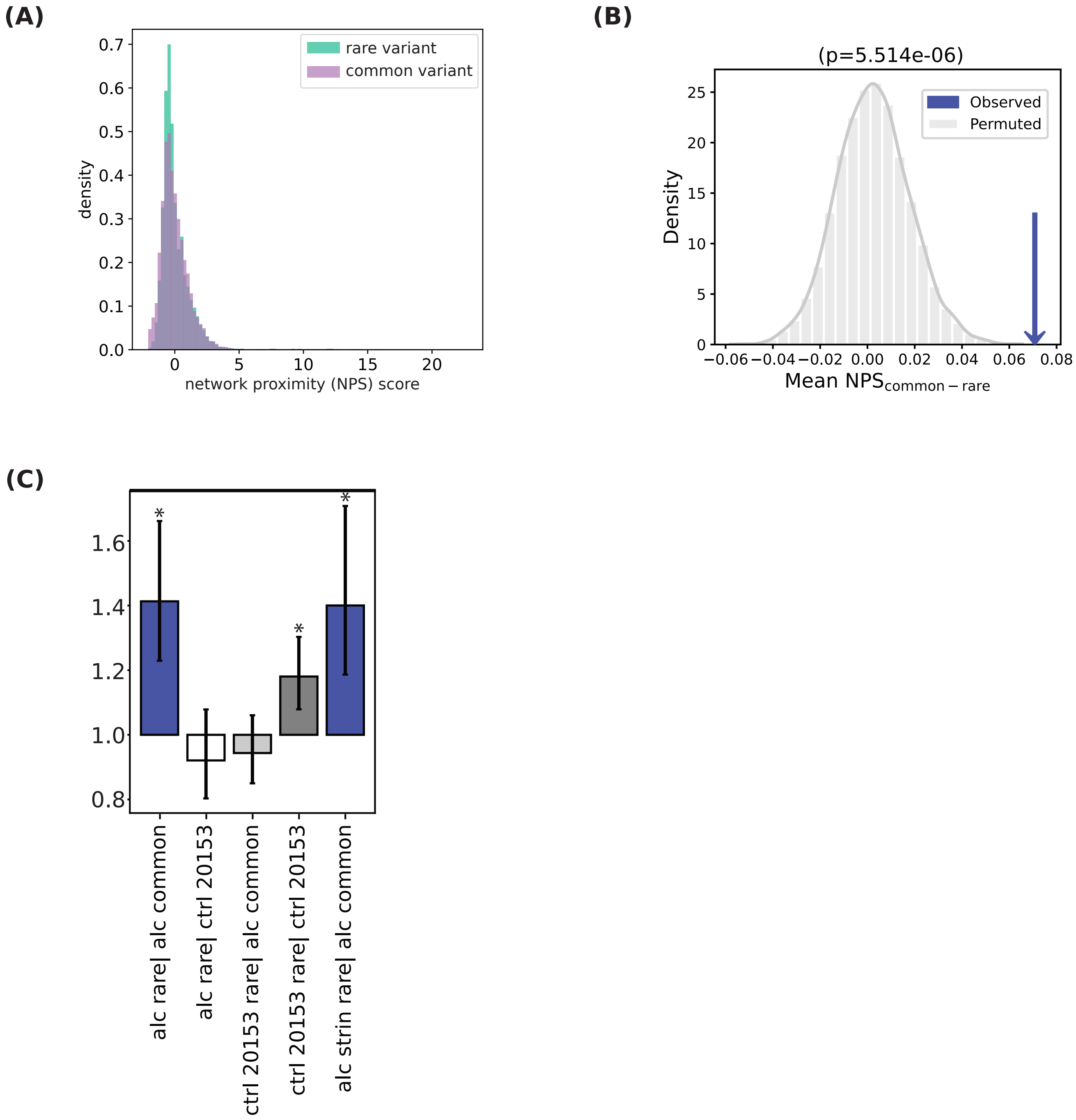

Supplemental Figure 2. Network colocalization of rare and common alcohol consumption seed genes. (**A**) Distribution of NPS_{common} and NPS_{rare} for all nodes in PCNet. (**B**) Observed (blue arrow) and expected mean $NPS_{comon\text{-}rare}$ for colocalization of common and rare seed genes, with significance assessed by Z-test. (**C**) The observed-to-expected ratio of colocalized network size from the following sources: alcohol consumption common and rare seed genes (left blue bar), negative control FEV1 (forced expiratory volume per second) common seed genes and alcohol consumption rare seed genes (white), FEV1 rare seed genes with alcohol consumption common seed genes (light gray), FEV1 rare and common seed genes (dark gray), and alcohol consumption stringent ($p_{SKAT\text{-}O}$<2.5x10-7) rare seed genes and common seed genes. Vertical bars indicate 95% confidence intervals. Significance calculated by Z-test, Bonferroni corrected. See Table S5 for additional controls.

bioRxiv preprint doi: https://doi.org/10.1101/2024.02.26.582195; this version posted March 1, 2024. The copyright holder for this preprint (which was not certified by peer review) is the author/funder, who has granted bioRxiv a license to display the preprint in perpetuity. It is made available under aCC-BY 4.0 International license.

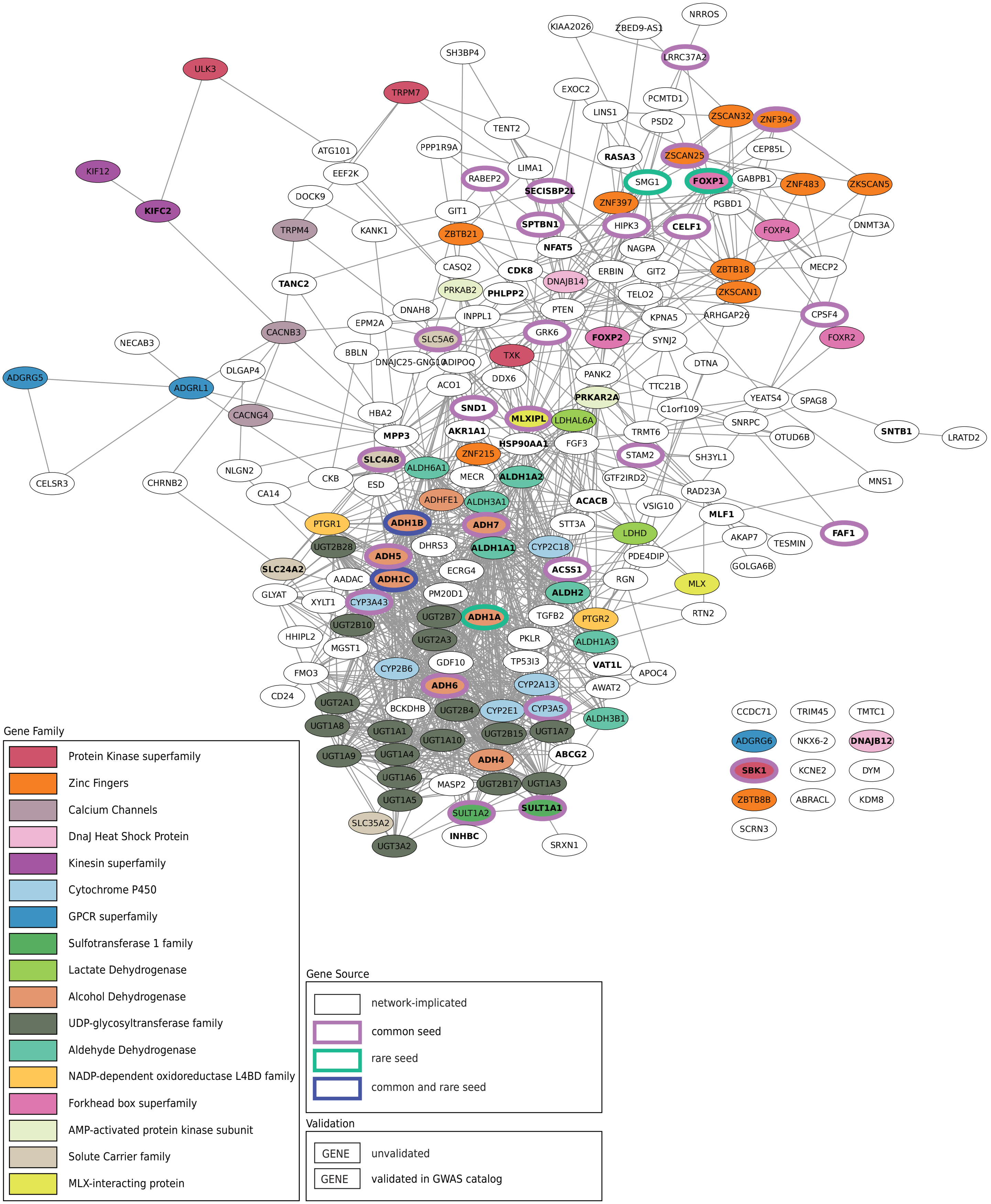

Supplemental Figure 3. Gene families in the alcohol consumption network. Subnetwork of PCNet including all genes proximal to both common and rare seed genes, as in Figure 3. Edges maintained from PCNet. Purple outlined nodes indicate common seed genes, green outlined nodes indicate rare seed genes, dark blue outlined nodes indicate seeds from both sources. Nodes with gene symbols in bold have previously been annotated in the GWAS catalog for alcohol related phenotypes. Gene families and functional groups were manually identified, and are indicated by color, as shown in legend. Only families with 2 or more genes present in the network were annotated.
